# Applications of 3D Printing Technology in Orthopedic Treatment

**DOI:** 10.1155/2021/9892456

**Published:** 2021-08-03

**Authors:** Xiaojun Duan, Ben Wang, Liu Yang, Anish R. Kadakia

**Affiliations:** ^1^Center for Joint Surgery, Southwest Hospital, Third Military Medical University (Army Medical University), Chongqing, China; ^2^Georgia Institute of Technology, Atlanta, USA; ^3^Northwestern University, Chicago, USA

Three-dimensional (3D) printing technology, also known as additive manufacturing (AM) or rapid prototyping (RP), is a special technique which could fabricate 3D models using computer-assisted design (CAD). It was firstly developed by a Japanese doctor forty years ago and initially used in manufacturing and industry [1]. During recent decades, with the development of manufacturing technology and materials science, 3D printing has also been used in some medical fields such as dentistry, maxillofacial surgery, and neurosurgery [2]. Application of 3D printing in orthopedics is also increasingly popular, mainly including preoperative planning, surgical guides, personalized implants, and customized prostheses [3, 4]. Individualized surgical treatment could be easily and accurately formulated under the aid of 3D printing and reduce the operation time and postoperative complications [5–7]. Depending on its unique advantages, 3D printing will lead a surgeon to precision medicine and provide patients with better treatment effects at lower cost [8, 9]. At present, the Chinese government, enterprises, universities, and institutes have invested a lot of resources in related research including printing technology, raw materials, and clinical applications and have made important progress. For example, our center uses 3D printing technology to manufacture implants of porous tantalum for clinical surgical treatment; in this special issue, a great majority of the submissions come from China, which report their latest developments in 3D printing. As the editorial team, we pay attention to some recent progressive research in 3D printing technology for orthopedic treatment. Below is a summary of these accepted articles.

The study by L. Kong et al. reported a set of articular spacer solutions using 3D printing technology in revision surgery for periprosthetic joint infection (PJI) after total knee arthroplasty. They compared the treatment effects between 3D printing spacer and static spacer in a retrospective study and stated that the 3D printing spacer group had less bone loss, less intraoperative blood loss, and greater knee function than the static spacer group. This technique effectively provides a new method to make accurate and personalized spacers in PJI and lower the rates of reinfection and complications.

The paper by Y. Du et al. evaluated the stability of the acetabular cup with different types of bone defects in total hip arthroplasty for developmental dysplasia of the hip (DDH) using the finite element analysis (FEA) model. The authors found that the diameter of the femoral ceramic head had no significant impact on the stability of the acetabular cup. When the uncoverage rates of the cup were less than 24.5%, the stability of the cup was satisfactory even without the use of screws. However, when the uncoverage rates were more than 24.5%, it was necessary to apply screws to improve the primary stabilization of the cup. Although their study is just based on the FEA model instead of clinical application, the results are still beneficial to the subsequent clinical study.

L. Yuan et al. retrospectively analyzed the bony resection accuracy during total knee arthroplasty (TKA) with patient-specific instrumentation (PSI) produced by 3D printing technology. They conducted full-length computed tomography (CT) for every patient and drafted detailed preoperative plans including the bony resection thickness. PSI was manufactured based on the CT data and operation plan. Each bone resected in the operation was also measured with CT to reconstruct the three-dimensional radiographs. The bone resection thickness was compared between the preoperative plan and intraoperative data to assess the resection accuracy in different bone sites. The results of this study show that PSI had a generally good accuracy during the femur and tibia bone resection in TKA.

X. Liu et al. evaluated the application of mixed reality (MixR) technology during transforaminal percutaneous endoscopic discectomy (TPED), and optical see-through head-mounted displays (OST-HMDs) were used to assist operation. They compared the difference of clinical effects between conventional TPED and MixR-assisted TPED and found that mixed reality (MixR) technology could significantly reduce the operation time and radiation exposure during the total operation procedure. This technology may be a powerful auxiliary tool for TPED but would increase the eye fatigue because of the application of OST-HMDs.

J. Kim et al. investigated whether the postcuring process could influence the dimensional accuracy and seating of 3D printing dental prostheses. A study stone model was designed and fabricated to verify this hypothesis. Results showed that the postcuring process significantly affected the fit and dimensional precision of 3D printing polymeric prostheses. They suggested that seating on the stone model was a better choice for minimizing the deformity of the dental prosthesis and reducing adverse effects during the postcuring process.

F. Gu et al. designed a three-dimensional printed patient-customized guiding template (3DGT) to increase the efficacy and safety of unicompartmental knee arthroplasty (UKA). Personalized guiding template could provide helpful assistance in several procedures of operation planning, intraoperative positioning, and osteotomy. This study concluded that 3DGT could shorten operation time, reduce surgical trauma, and promote recovery.

P. Honigmann et al. presented the first inhospital 3D printed scaphoid prosthesis using polyetheretherketone (PEEK) biomaterial via fused filament fabrication (FFF), one of the 3D printing technologies. The surface of this medical grade PEEK prosthesis did not show “FFF stair-stepping” phenomenon, which was usually common in the industrial grade scaphoid prosthesis. The biocompatible and implantable polymers such as PEEK applied in 3D printing could offer great potential in the treatment of complex joint damage in the hospital environment.

M. Keller et al. reviewed the latest practical application of 3D printing in hand surgery and introduced the most common printing techniques and some materials. They provided a useful overview of the 3D printing technology applied in numerous aspects such as surgical guides, personalized implants for bone defects, customized splints, and preoperative plan. The authors hold the opinion that orthopedics, especially hand surgery, will benefit from 3D printing in the near future.

L. Cheng et al. retrospectively investigated the utilization and feasibility of 3D printing technology for core decompression in patients with osteonecrosis of the femoral head (ONFH). The operation process went well and consumed less time than traditional methods with the aid of personalized guide plates and reduced the usage of intraoperative X-ray fluoroscopy. The results indicated that 3D printing had several advantages of improving efficiency, being more convenient, and accurate positioning.

C. Zhang et al. revealed the efficacy of arthroscopy in treating bone cysts of the foot and ankle combined with 3D printing individualized guides. Better VAS score and AOFAS score and less intraoperative bleeding were displayed in patients with the assistance of 3D printing. It is concluded that 3D printing could significantly help surgeons to fast and smoothly establish a portal in arthroscopic ankle surgery.

J. Fu et al. reconstructed the acetabular bone defect in a swine model to evaluate the bone ingrowth, biomechanics, and matching degree of the 3D printed porous prosthesis. Based on the results, the authors found that the 3D printed porous augments showed great porosity and pore size and had magnificent stiffness and elastic modulus. The anatomical matching extent was excellent, which could enhance the stability of the porous prosthesis. Although this study was conducted in minipigs, it displayed the great potential of 3D printed porous augment in the treatment of clinical severe acetabular bone defects.

Y. Mao et al. compared the clinical effects of 3D printed patient-specific instrumentation (PSI) with conventional surgical techniques in medial open wedge high tibial osteotomy (MOWHTO). The results of this prospective comparative study showed that 3D printed PSI had significantly lower correction errors in terms of mFTA and mMPTA and demanded shorter duration and less radiation exposure. They concluded that 3D printing technique could be recommended as an effective assistant for MOWHTO in the treatment of varus because of its accuracy and effectiveness.

W. Peng et al. reported an entirely anatomically conforming pelvic prosthesis for pelvic reconstruction. Pelvic tumor is a complex disease due to the vascular invasion of tumor issue, and most of the patients suffering from pelvic tumor undergo the surgery of tumor resection and hemipelvic replacement. The authors showed that 3D-printed prosthesis was of value for patients with complex pelvic tumors.
